# First deployment of artificial intelligence recommendations in orthopedic surgery

**DOI:** 10.3389/frai.2024.1342234

**Published:** 2024-01-31

**Authors:** Farid Al Zoubi, Koorosh Kashanian, Paul Beaule, Pascal Fallavollita

**Affiliations:** ^1^School of Electrical Engineering and Computer Science, University of Ottawa, Ottawa, ON, Canada; ^2^Division of Orthopedic Surgery, Ottawa Hospital Research Institute, Ottawa, ON, Canada; ^3^Interdisciplinary School of Health Sciences, University of Ottawa, Ottawa, ON, Canada

**Keywords:** machine learning, healthcare system, operating room efficiency, orthopedic surgery, prescriptive analytics

## Abstract

Scant research has delved into the non-clinical facets of artificial intelligence (AI), concentrating on leveraging data to enhance the efficiency of healthcare systems and operating rooms. Notably, there is a gap in the literature regarding the implementation and outcomes of AI solutions. The absence of published results demonstrating the practical application and effectiveness of AI in domains beyond clinical settings, particularly in the field of surgery, served as the impetus for our undertaking in this area. Within the realm of non-clinical strategies aimed at enhancing operating room efficiency, we characterize OR efficiency as the capacity to successfully perform four uncomplicated arthroplasty surgeries within an 8-h timeframe. This Community Case Study addresses this gap by presenting the results of incorporating AI recommendations at our clinical institute on 228 patient arthroplasty surgeries. The implementation of a prescriptive analytics system (PAS), utilizing supervised machine learning techniques, led to a significant improvement in the overall efficiency of the operating room, increasing it from 39 to 93%. This noteworthy achievement highlights the impact of AI in optimizing surgery workflows.

## 1 Introduction

The application of artificial intelligence (AI) in healthcare has seen remarkable advancements since its inception, encompassing various areas such as diagnosis, genetics, prognosis, and drug discovery (Bohr and Memarzadeh, 2020). Despite the vast potential, the predominant focus has often been on the clinical aspect of AI, specifically targeting patient-centered applications rather than addressing the broader spectrum of healthcare processes (Maier-Hein et al., 2022).

A noticeable trend in recent developments revolves around the competitive drive to enhance surgical procedures through the integration of AI. Robotic technologies, in particular, have garnered significant attention among researchers and AI inventors, reflecting a concentrated effort to revolutionize surgical practices (Nwoye et al., 2023).

In contrast, a fewer body of work has delved into the non-clinical facets of AI, concentrating on leveraging data to enhance the overall healthcare system and operating room efficiency.

This includes initiatives aimed at improving team efficiency (Al Zoubi et al., 2022), optimizing patient appointment scheduling (Erekat et al., 2020), and predicting overall procedural durations either partially (Bartek et al., 2019; Schiele et al., 2021; Strömblad et al., 2021) or comprehensively (Al Zoubi et al., 2023).

Despite these advancements, a notable gap exists in the literature pertaining to the deployment and outcomes of AI solutions (Jiang et al., 2021). As of now, there is a lack of published results showcasing the practical implementation and effectiveness of AI solutions in areas beyond the clinical realm, particularly in the field of surgery.

Some studies that have applied AI in healthcare have examined the obstacles that may hinder progress in this field. These challenges might stem from the interdisciplinary nature of AI solutions in healthcare (Safavi et al., 2019), a limited availability of interpretable machine learning models, resistance from medical unions and associations, extended processing times (Bertsimas et al., 2022), and the complexities associated with decision-making and bureaucratic processes involving multiple stakeholders (Hu et al., 2021).

Within the realm of non-clinical strategies aimed at enhancing operating room efficiency, we characterize OR efficiency as the capacity to successfully perform four uncomplicated arthroplasty surgeries within an 8-h timeframe. In this Community Case Study, we disclose the outcomes arising from the incorporation of AI recommendations at The Ottawa Hospital (TOH), Ottawa, Canada leading to a substantial enhancement in the overall efficiency of the arthroplasty operating room, elevating it from 39 to 93%. This remarkable accomplishment was realized by deploying a prescriptive analytics system (PAS) that utilizes supervised machine learning techniques to generate benchmarks specifically tailored for arthroplasty surgery workflows.

## 2 Methodology

### 2.1 Problem statement

**Figure 1:**
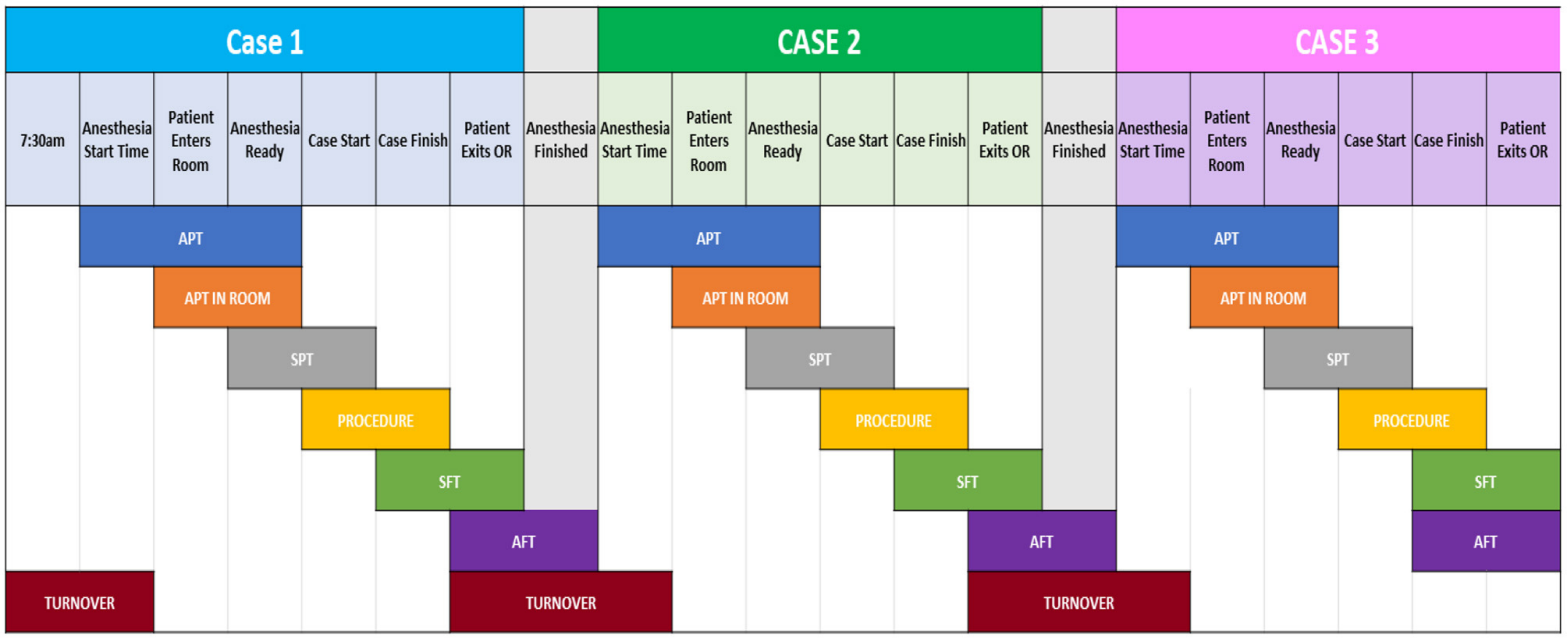
A breakdown of each step-in arthroplasty, along with the transition between two stages (turnover), is outlined. The upper section indicates the stage markers for a case. Only showing three out of four cases a day.

Our institution has a specialized orthopedic operating room dedicated to 4-joint arthroplasty procedures. This facility is designed specifically for high-volume arthroplasty surgeries, encompassing both partial and complete joint replacements. It accommodates four procedures each day from Monday to Friday, with the exception of Wednesdays. The procedural workflow comprises six stages: Anesthesia Preparation, patient positioning, the surgical procedure itself, patient exiting the room and turnover, and the final stage, as illustrated in Figure 1 (Al Zoubi et al., 2022). The scientific names and abbreviations for these stages are provided in Table 1.

**Table 1:** Medical names and acronyms for arthroplasty phases.

**Time metrics**
Anesthesia preparation time (APT)
Anesthesia finish time (AFT)
Surgical preparation time (SPT)
Surgery finish time (SFT)
Turnover
Surgery (procedure) time

A successful day in this arthroplasty operating room is defined as completing all four procedures within the allocated time span of 8 h, which falls between 7:30 am and 3:30 pm. The Surgical Success Rate (SSR) serves as the metric to gauge the percentage of successful days. The initial SSR was notably low at 39%, contributing to an annual overtime cost of ~$570,000 for our institution.

### 2.2 Previous work

Recently, we proposed a comprehensive solution to address this challenge, a data-driven, Machine Learning (ML)-based, prescriptive analytics system (PAS). This decision support system not only predicts the likelihood of a particular day being successful based on temporal variables but also monitors each stage of the procedure in real-time. It adjusts its predictions as needed and provides actionable suggestions at each stage to enhance the likelihood of success (Al Zoubi et al., 2022).

The journey toward creating the PAS, aimed at enhancing the Surgical Success Rate (SSR) of high-volume arthroplasty surgeries, began by implementing Descriptive Analytics on retrospective data of ~5,000 surgeries (Al Zoubi et al., 2024). In addition to providing actionable insights, this phase allowed us to categorize the surgical recorded parameters into patient metrics, team metrics, and time metrics. For each group of metrics, we developed a framework that serves as a decision support system to enhance SSR independently.

As the fundamental research progressed into the Predictive Analytics stage, we proposed the adoption of a Workflow/Time monitoring framework (WTMF; Al Zoubi et al., 2022), for the transformation of our PAS. The WTMF effectively aligns with our objective of improving SSR through improving the overall team efficiency. The conversion of WTMF into a prescriptive system involved several stages, such as conducting what-if scenarios, generating benchmarks, and identifying a set of actions and recommendations, which were determined through multidisciplinary positive deviance seminars (Gold et al., 2022). The Machine Learning (ML) engine in our Predictive Analytics System (PAS) predominantly relies on the decision tree technique. This is true for both predicting the Surgical Success Rate (SSR) and generating dynamic benchmarks. Additionally, it has the capability to incorporate various other supervised learning classifiers specifically for SSR prediction. The imperative for an interpretable ML model is highlighted, particularly in the context of benchmark generation. We elucidated and compared several ML techniques in our previous work to underscore this necessity.

Ultimately, working under the assumption of utilizing the WTMF, we conducted calculations to determine both cost savings and the possibility of adding an extra 5th joint surgery on days when the initial four joint surgeries were successfully completed on time, all in an 8-h window (Al Zoubi et al., 2023).

The central focus of this study revolves around the comparison between theoretical concepts and practical outcomes. We assess the performance of our algorithms and evaluate the efficacy of implementing the WTMF into an integrated PAS on prospective patient surgeries.

**Figure 2:**
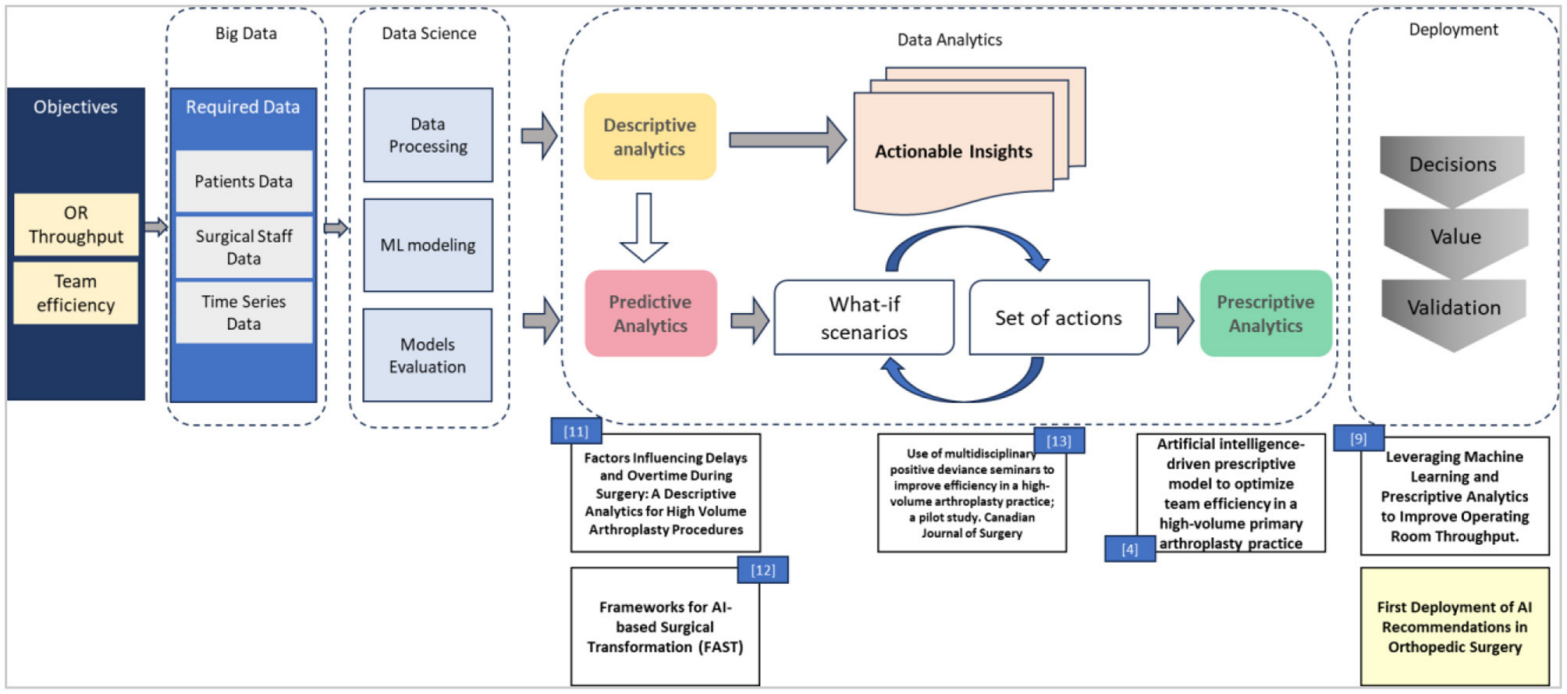
Design and deployment roadmap of AI-driven recommendations to improve the surgical success rate.

The overall journey of designing and implementing our AI-driven solution to improve the SSR is summarized in Figure 2. The boxes below display the titles of published articles linked to each respective phase, identified by their corresponding reference numbers in the bibliography.

### 2.3 Validation on arthroplasty surgeries—patient dataset

The PAS was implemented and validated at TOH's Riverside Campus, involving a team of seven arthroplasty surgeons, along with a group of nurses and anesthesiologists. The surgical procedures were conducted weekly, utilizing two operating rooms each week, spanning ~23 Saturdays in 2023. Table 2 offers a concise overview of crucial statistical data sourced from 228 patients who underwent hip and knee replacement surgeries.

**Table 2:** Statistical summary of the 228 patient surgeries used in the validation of the PAS.

Surgeries	228
Number of surgeons	7
Number of nurses team	43
Number of anesthesiologist	13
Female patients	130
Male patients	98
Average age	65.3 ± 8.4
Average Body Mass Index (BMI)	28.7 ± 5.5
Average American Society of Anesthesiologists classification (ASA)	2 ± 0.7
Average number of surgeries/OR/day	4.95 ± 0.6

Additionally, the surgical team received pre-surgery targeted benchmarks and recommendations like the ones in our previous work 4. Further elaboration on the dataset and its potential impact on the outcomes is provided below.

#### 2.3.1 Patient age, BMI, and gender

The demographic characteristics of the dataset reveal that the surgeons performed operations on individuals of advanced age and with high BMI. The results of descriptive analytics indicated that age and BMI have minimal influence on the SSR (Al Zoubi et al., 2024). However, in our Saturday data, a notable gender disparity was observed, with surgeons operating on ~25% more females than males. This discrepancy is expected to have an impact on achieving a higher SSR, as females tend to have a shorter operating time by ~5 min when compared to males. For this dataset, this observation remains consistent with our previously discussed conclusions in Al Zoubi et al. (2024).

#### 2.3.2 Patient ASA

Performing surgeries on patients with pre-existing medical conditions raises the likelihood of complications and consequently, the potential delay of the 4th joint surgery. The Saturday data reveals an average ASA score of 2, with a standard deviation of 0.7, indicating that many patients have underlying medical conditions.

#### 2.3.3 Surgeon demographics

**Figure 3:**
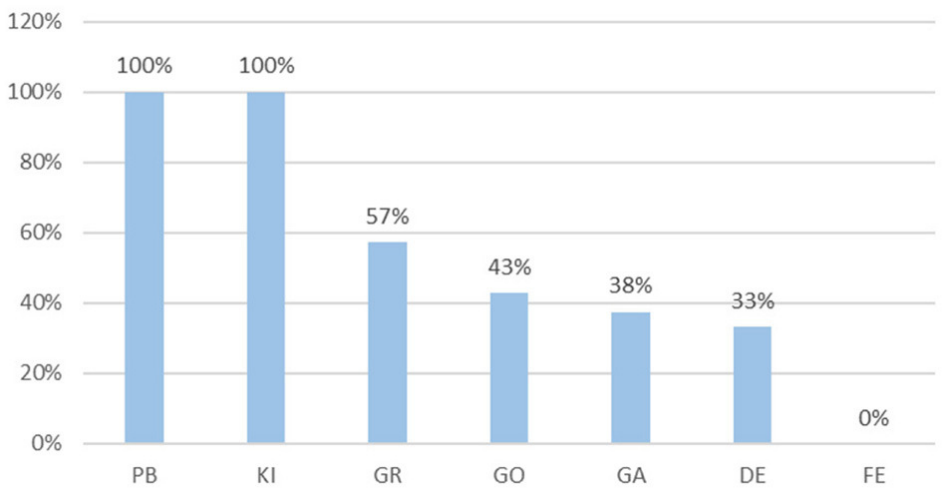
SSR per surgeon.

The quantity of surgeries performed by each surgeon ranges from a maximum of 40 surgeries to a minimum of 29 per surgeon. The SSR for each surgeon is presented in Figure 3.

Furthermore, we conclude that the surgeon's experience does not exhibit a direct correlation with SSR. For instance, even though “GO” possesses over a decade of experience in hip surgeries, his SSR is only 43%. This finding aligns with the conclusion reached in our earlier publication (Al Zoubi et al., 2024).

### 2.4 Outcomes and observations

Table 3 showcases the central tendency values for the benchmark metrics observed during the 23 Saturdays.

**Table 3:** Central tendencies for benchmarks observed on Saturday surgeries.

**Metric**	**APT**	**SPT**	**Procedure**	**AFT**	**Turnover**
Minimum	01:00	00:00	30:00	01:00	02:00
Maximum	41:00	26:00	04:00	28:00	45:00
Mean	11:45	09:05	56:26	04:05	20:47
Median	11:00	09:00	55:00	04:00	20:00
Standard deviation	0.4%	0.3%	1.5%	0.2%	0.5%

To facilitate comparisons, we have included the mean values of these metrics in Table 4, derived from the earlier generated machine learning output (MLO) benchmarks in Al Zoubi et al. (2023). When comparing with the chosen benchmarks, it becomes evident that there is a notable improvement in SSR. The calculated SSR for Saturday surgeries reached 93%, falling between the MLO values of 91 and 100%. Interestingly, it appears that a procedure that is 3$\frac{1}{2}$ min faster has a more substantial impact on SSR than the cumulative 2-min slowdown in both turnover and APT when compared to MLO-Fast Performance. This supports our earlier conclusion in Al Zoubi et al. (2022), indicating that a 1-min difference in a specific stage can have a significantly larger impact on the outcome compared to a 1-min difference in another stage due to non-linearity.

**Table 4:** Machine learning output (MLO) benchmarks compared to Saturday surgery practices.

**Benchmarks scenario**	**APT**	**SPT**	**Procedure**	**AFT**	**Turnover**	**SSR**
MLO-optimal performance	7:00	7:00	62:30	7:00	20:00	100%
Saturday clinical practices	11:45	9:05	56:26	4:05	20:47	**93%**
MLO-fast performance	10:00	12:00	60:00	4:00	20:00	91%
MLO-baseline	10:30	20:00	71:30	20:30	21:30	77%
MLO-fair performance	18:30	20:00	71:30	20:30	21:30	64%
MLO-poor performance	18:00	20:00	77:00	8:00	31:00	0%

Bold value indicates the achieved SSR value leveraging the PAS system.

It's crucial to keep in mind that before the implementation of the PAS, the overall SSR at The Ottawa Hospital consistently remained below 39% for arthroplasty surgeries, regardless of the surgeon involved, and including the positive deviance surgeon whose SSR was 68% (Jung et al., 2020). However, with the implementation of PAS, the SSR has surged to 93%. This achievement significantly surpasses the suggested Machine Learning Output (MLO) baseline of 77%, not to mention the pre-PAS SSR, which stood at a mere 39%.

## 3 Discussion

This Community Case Study aimed to evaluate the actual improvement of TOH's arthroplasty operating room efficiency; thus, we have opted to assess and validate the performance of our designed prescriptive analytics system originating from one of the three AI-driven frameworks we had previously published, namely the WTMF.

The initial implementation of the PAS occurred at TOH and involved a collective effort from surgeons, nurses, and anesthesiologists who voluntarily chose to extend their work to Saturdays, aligning with the Ontario government's initiative to increase surgical capacity. TOH has allocated two operating rooms specifically for hip and knee replacement surgeries on Saturdays.

We had anticipated that upon the initial implementation of the WTMF, the surgical team would strive to reach the recommended baseline SSR of 77%. To our surprise, the team achieved a 93% success rate in completing their fourth joint case within an 8-h window. Furthermore, they were able to accommodate an additional case 52% of the time within the same 8-h window (Al Zoubi et al., 2023). Nevertheless, certain considerations must be considered regarding our results.

First, performing surgeries on weekends and specifically on Saturdays, offers the advantage of minimal interruptions from other hospital entities or phone calls, creating a conducive environment for surgeries. This could have had a positive impact on the outcomes. However, in our previous study in Al Zoubi et al. (2024), we demonstrated that the day of the week itself influences SSR. We found that SSR tends to be lower the closer the surgical day is to the weekend (since people look forward to ending their week quickly). This contrast in the impact of the day of the week, with potential positive and negative effects, makes it challenging to definitively determine its true influence on SSR. However, the day of the week does indeed have a distinct impact compared to surgeries conducted on weekdays.

The second factor pertained to calculating turnover times for the first case. During regular workweeks, nurses' shifts commence at 7:30 am, and the time of the first patient's entry into the operating room varies depending on team efficiency. It is well-established that the earlier the patient enters the room, the sooner the day concludes. However, on Saturdays, due to the absence of nursing union policies dictating start times, surgeons have the flexibility to commence surgeries at their discretion. To ensure data comparability, the 8-h time stamp needed to commence when the patient entered the room, as the time prior to the entry of the first case could fluctuate based on the “shift start” time.

The third consideration arises from the fact that one of the surgeons had previously been identified as an individual demonstrating positive deviance (PD) during the seminars. This prior acknowledgment could potentially introduce a positive bias to the outcomes. However, the results indicated that another surgeon achieved the same SSR as the PD surgeon, leaving us uncertain as to whether the PD recognition had influenced the data or if this level of success could be achieved under normal circumstances. It's important to note that there were no positive deviance seminars conducted specifically for this group of surgeons on Saturdays; the established benchmarks were simply communicated amongst team members as well as the culture of working together.

The fourth challenge we encountered was the manual implementation of the PAS, which involved fixed benchmark values being communicated to the practitioners. In case of any delays, surgeons had to refer to suggested actions from previous positive deviance seminars to manage the subsequent stages. Conversely, if we are to transform the PAS into a real-time decision support software, it will have the capability to generate adjusted benchmarks for upcoming stages through AI, which can assist in completing them on time or even ahead of schedule. The anticipated flexibility provided by the real-time PAS is expected to yield superior outcomes.

Finally, as explained in the preceding section there are 25% fewer male patients compared to female patients. This factor may have had a positive effect on the outcomes.

## 4 Conclusions

The transformed WTMF into PAS was put into action within a real clinical setting for validation purposes. This implementation spanned 228 surgical cases, demonstrating the effectiveness of the algorithms, resulting in improved team efficiency, and increased operating room throughput. This is evident through the attainment of a 93% SSR and the ability to accommodate an additional case every other week, all without incurring any additional costs.

To our knowledge, this is the first experience in deploying AI recommendations in orthopedic surgery. The work done to improve SSR in arthroplasty surgeries can be extended to other types of high-volume surgeries as well.

Two alternative frameworks previously published Surgical Team Scheduling Framework (STSF) and Patients Schedular Framework (PSF), can likewise be converted into PAS using the same techniques and methodologies employed for the WTMF. This transformation can be accomplished by creating what-if scenarios, gathering actions for each scenario, and leveraging both multidisciplinary and interdisciplinary approaches. However, the implementation of the STSF may be controversial since at its premise, the AI recommendation is demanding specific individuals to possibly compromise in certain work activities to increase in the SSR.

Lastly, we have the desire to investigate more critical and detailed factors, such as the behavior of the staff within the operating room using cameras (Jung et al., 2020), or the potential impact of the machine and instrument vendors on operational delays. Additionally, we would like to analyze data related to drugs and anesthesia types to understand their impact on early wake-up occurrences. Another possibility is merging multiple frameworks. However, the availability of data becomes a limiting factor in exploring these aspects thoroughly.

## Data availability statement

Requests to access these datasets should be directed to falzo100@uottawa.ca.

## Ethics statement

The studies involving humans were approved by Ottawa Health Science Network Research Ethics Board (OHSN-REB). The studies were conducted in accordance with the local legislation and institutional requirements. Written informed consent for participation was not required from the participants or the participants' legal guardians/next of kin in accordance with the national legislation and institutional requirements.

## Author contributions

FA: Conceptualization, Data curation, Formal analysis, Investigation, Methodology, Resources, Software, Validation, Visualization, Writing – original draft, Writing – review & editing. KK: Data curation, Writing – review & editing. PB: Conceptualization, Writing – review & editing. PF: Conceptualization, Methodology, Project administration, Resources, Supervision, Writing – review & editing.
